# Editorial: Emerging approaches for enhancing nutritional quality of berries

**DOI:** 10.3389/fpls.2023.1227185

**Published:** 2023-06-23

**Authors:** Luca Mazzoni, Iraida Amaya, Gabriella De Lorenzis, Alba N. Mininni

**Affiliations:** ^1^ Department of Agriculture, Food, and Environmental Sciences, Università Politecnica delle Marche, Ancona, Italy; ^2^ Breeding and Biotechnology, Andalusian Institute for Research and Training in Agriculture, Fisheries, Food and Ecological Production (IFAPA), IFAPA Centre of Málaga, Malaga, Spain; ^3^ Department of Agricultural and Environmental Sciences, Università degli Studi di Milano, Milano, Italy; ^4^ Department of European and Mediterranean Cultures, Environment and Cultural Heritage, Università degli studi della Basilicata, Matera, Italy

**Keywords:** QTL, genotype, polyphenols, antioxidant, gene expression

Consumers today are increasingly aware of the link between food and health, recognizing that certain food components can positively impact physiological processes and help prevent chronic diseases. Plant-based products are widely consumed worldwide, making them a promising avenue for improving population health. Developing new plant-based foods with enhanced nutritional properties, achieved through conventional and molecular breeding techniques, can have a significant impact. Berries, known for their appealing taste, color, and aroma, are natural sources of various nutrients and are important for a balanced diet. Researchers are actively exploring new methodologies and technologies to increase genetic variability, expedite breeding processes, and facilitate the combination of desired traits in new plant varieties. This Research Topic invited excellent Original Research and Review articles focusing on enhancing the nutritional quality of berries.

Three of the six collected original research papers focus on identifying genetic and biochemical determinants affecting the accumulation of bioactive compounds in blueberries. Mengist et al. investigate the genetic mechanisms controlling health-promoting compounds such as anthocyanin and chlorogenic acid, and their relationship with important fruit quality traits like acidity and total soluble solids (TSS). Their study employed a population of 196 F_1_ lines derived from the cross of highbush cultivars ‘Draper’ and ‘Jewel’ to dissect quantitative trait locus (QTL) for these traits over three years. An environmentally stable QTL for fruit acidity on chromosome 3 and another for TSS on chromosome 10, also detected in other studies, represent exciting targets for candidate gene analyses and marker-assisted selection. Interestingly, besides the observed correlation between glycosylated anthocyanins and TSS, a common QTL on chromosome 8 might indicate a common control point for these traits. In contrast, a QTL for chlorogenic acid on chromosome 2 spans the same region of a QTL for acylated anthocyanins but is located in different haplotypes, hindering the parallel selection of positive-effect haplotypes. A major QTL for anthocyanidin glycosylation is reported on chromosome 4 and collocates with a *flavonoid 3-O-glucosyltransferase*. Furthermore, the authors show significantly higher expression in blueberry lines with high-glycosylated anthocyanins, indicating that it might be the gene underlying the QTL. In the article from Montanari et al., a different mapping population was used, derived also from two highbush cultivars, ‘Nui’ and ‘Hortblue Petite’, and major and minor effect QTLs for anthocyanin content and composition are reported. Consistent with the results of Mengist et al., two major QTLs for acylated anthocyanins and glycosylated anthocyanins were detected on chromosomes 2 and 4 in the two analyzed seasons. Common candidate genes to Mengist et al. and other *MYB* transcription factors and *dihydroflavonol 4-reductases* were identified in QTL intervals, representing exciting candidates for future analyses. These two studies highlight that MAS could be feasible for particular anthocyanins and bioactive compounds controlled by major-effect and stable QTLs.

The third study related to blueberry by Cocetta et al. aimed to investigate the effects of pre-harvest benzothiadiazole (BTH) treatments on fruit quality, ascorbic acid oxidation and recycling metabolism, and phenolic compounds accumulation, during the development and ripening of two selected blueberry cultivars (*Vaccinium corymbosum* cv ‘Brigitta’ and ‘Duke’). The most significant results showed that BTH application had no marked effects on fruit quality parameters, but could positively impact fruit bioactive compounds levels, affecting the activities of all enzymes of the recycling pathway of ascorbic acid and increasing polyphenols accumulation in fruit, partly depending on cultivar and ripening stage. The study suggests that BTH application could effectively trigger a stress response, as increased ascorbic acid contents reflected the short-term reaction of the plant, resulting in activation of the secondary metabolic pathway that leads to the accumulation of health-promoting compounds (including polyphenols, anthocyanins, flavonoids), directly involved in the improvement of the nutraceutical value of the product. These findings provide significant insights into the potential application and role of BTH and the responses which are modulated differently according to genotypes, contributing to help breeding programs aimed to develop cultivars with optimal traits in terms of health-promoting components and quality attributes.

Moving to other berry species, Milella et al. investigated the association between the phenolic profile, antioxidant, and anticancer activities of recently discovered table grape genotypes from Apulia, Italy. The research demonstrated a positive correlation between the phenolic content of the grapes and their antioxidant and anticancer activities. Moreover, the grape genotypes exhibited varying levels of antioxidant and anticancer activities, with some genotypes showing higher activities than others. The study suggests that the phenolic compounds found in grapes may be responsible for their beneficial health effects and that the development of new grape varieties with high phenolic content could lead to the production of grapes with increased antioxidant and anticancer activities. The findings provide significant insights into the potential health benefits of grapes and underscore the significance of developing new grape varieties with high phenolic content for improving human health outcomes.

The impact of genotype, together with terroir, seasonality, and farming systems (conventional and organic) was also investigated on functional traits of two tomato cultivars (San Marzano, Round, and Roma, Long) by Rocchetti et al. The study demonstrated through a multivariate statistical approach that the phytochemical profile of tomato fruits was hierarchically more affected by the origin and the cultivar than by the farming system, identifying the pedo-climatic conditions and the genotype as the main determinants for the functional quality of tomato. The achieved results showed that the intricate interaction of cultivar and environment, followed by agronomic factors, may play a key role in shaping the potential health-promoting effects and associated benefits of vegetables, such as through the metabolomic profile of tomato fruits and, consequently, their antioxidant and enzyme inhibition capacities. These findings provide significant insights into the relevant contribution of several factors (cultivars, terroir, seasonality) on the functional properties and quality of organic or conventional tomatoes in a broader framework of food safety aspects and sustainability issues.

Finally, Yang et al. investigated the regulation of citric acid metabolism during the late developmental stages of strawberry fruit. The study identified that the genes FaGAPC2/FaPKc2.2 and FaPEPCK were highly expressed during the late developmental stages of the fruit and were involved in the regulation of citric acid metabolism. These genes were found to be regulated by abscisic acid (ABA) and sugars, which play critical roles in fruit development and ripening. The study suggests that the regulation of citric acid metabolism in the late developmental stages of strawberry fruit is complex and involves several genes and regulatory pathways. These findings have significant implications for the development of new strawberry varieties with improved fruit quality and extended shelf life. The study provides valuable insights into the molecular mechanisms underlying the regulation of citric acid metabolism in strawberry fruit and highlights the potential of genetic engineering approaches to enhance fruit quality and post-harvest storage.

The Original research studies submitted to this Research Topic underlined the importance of different approaches for the enhancement of nutritional quality in different berry species. The application of molecular, agricultural, and biochemical tools to increase the presence of nutritional molecules in fruits are all valuable and exploitable solutions for the obtainment of new healthier berries, shortening the breeding processes and facilitating the combination of traits of interest in the new progeny.

## Author contributions

All authors listed have made a substantial, direct, and intellectual contribution to the work and approved it for publication.

